# Prediction of 30-day, 90-day, and 1-year mortality after colorectal cancer surgery using a data-driven approach

**DOI:** 10.1007/s00384-024-04607-w

**Published:** 2024-02-29

**Authors:** Karoline Bendix Bräuner, Andi Tsouchnika, Maliha Mashkoor, Ross Williams, Andreas Weinberger Rosen, Morten Frederik Schlaikjær Hartwig, Mustafa Bulut, Niclas Dohrn, Peter Rijnbeek, Ismail Gögenur

**Affiliations:** 1 Center for Surgical Science, Zealand University Hospital, Køge, Lykkebækvej 1, 4600 Køge, Denmark; 2 University of Copenhagen, The Faculty of Health Science, Blegdamsvej 6, 2200 Copenhagen N, Denmark; 3 Department of Medical Informatics, Erasmus University Medical Center, Doctor Molewaterplein 40, 3015 GD Rotterdam, Holland, Netherlands; 4 Department of Surgery, Copenhagen University Hospital, Herlev & Gentofte, Borgmester Ib Juuls vej 1, 2730 Herlev, Denmark

**Keywords:** Colorectal cancer, Machine learning, Prediction model, Mortality, Postoperative

## Abstract

**Purpose:**

To develop prediction models for short-term mortality risk assessment following colorectal cancer surgery.

**Methods:**

Data was harmonized from four Danish observational health databases into the Observational Medical Outcomes Partnership Common Data Model. With a data-driven approach using the Least Absolute Shrinkage and Selection Operator logistic regression on preoperative data, we developed 30-day, 90-day, and 1-year mortality prediction models. We assessed discriminative performance using the area under the receiver operating characteristic and precision-recall curve and calibration using calibration slope, intercept, and calibration-in-the-large. We additionally assessed model performance in subgroups of curative, palliative, elective, and emergency surgery.

**Results:**

A total of 57,521 patients were included in the study population, 51.1% male and with a median age of 72 years. The model showed good discrimination with an area under the receiver operating characteristic curve of 0.88, 0.878, and 0.861 for 30-day, 90-day, and 1-year mortality, respectively, and a calibration-in-the-large of 1.01, 0.99, and 0.99. The overall incidence of mortality were 4.48% for 30-day mortality, 6.64% for 90-day mortality, and 12.8% for 1-year mortality, respectively. Subgroup analysis showed no improvement of discrimination or calibration when separating the cohort into cohorts of elective surgery, emergency surgery, curative surgery, and palliative surgery.

**Conclusion:**

We were able to train prediction models for the risk of short-term mortality on a data set of four combined national health databases with good discrimination and calibration. We found that one cohort including all operated patients resulted in better performing models than cohorts based on several subgroups.

**Supplementary Information:**

The online version contains supplementary material available at 10.1007/s00384-024-04607-w.

## Introduction

Colorectal cancer (CRC) is the second leading cause of cancer-related death and the third most common malignant neoplastic disease worldwide with an annual incidence of 1.8 million new cases [1, 2]. The cornerstone of curative treatment of CRC is surgery, which is known to have a risk of postoperative complications of up to 47.4%, 14.4% major complications, and mortality of 3.3% in the postoperative period [3]. The past decades have seen vast progress in reducing mortality after colorectal cancer surgery [4]; treatment and complication rates may improve further by personalizing treatment based on each patient’s individual challenges.

Approximately one third of patients diagnosed with CRC can be considered frail due to age, comorbidity, functional capacity, and lifestyle factors [5, 6], and these patients face a higher risk of postoperative mortality [7]. Several studies suggest that frailty is an independent factor of increased mortality and complications after surgery and that identifying frail patients and optimizing their trajectory in relation to surgery can reduce the risk of postoperative adverse outcomes [5, 8, 9]. Similarly, to the variation in frailty, there is large heterogeneity in terms of age, comorbidities, nutritional deficiencies, and histopathological tumor variation between patients with CRC [3, 10, 11]. Several interventions exist to target these challenges such as iron infusions for anemic patients [12, 13], prehabilitation [14–16], medical nutrition therapy [17], optimization of medication, and even different neoadjuvant treatment strategies depending on the tumor are under development [18]. In order to individualize a patients’ treatment based on their risk profile, a clinician requires knowledge of the personalized risk of this patient. Therefore, a tool that can provide these estimates would aid in the process of treatment planning.

With this study, we aimed to develop prediction models using a data-driven approach on preoperative data from multiple nationwide health databases focusing on mortality within 30 days, 90 days, and 1 year after CRC surgery, which in the future could be used for patient stratification and personalized treatment.

## Methods

### Data sources

Data were retrieved from four Danish observational health databases with nationwide coverage. These are the Danish Colorectal Cancer Group database (DCCG) including information about the patient’s CRC history with a 95–99% coverage from 2001 to 2019 [19, 20], the Danish National Patient Register (DNPR) with trajectory data from all contacts with the secondary healthcare sector from 1976 to 2019 [21], Register of Laboratory Results for Research (RLRR) containing biochemical and microbiological laboratory results from 2013 to 2019 [22], and the National Prescription Registry (NPR) containing information about claimed drug prescriptions from 1994 to 2019 [23].

Access to Danish observational health databases for research does not require ethical approval. This study was approved for Region Zealand under the record number REG-102–2020 [24].

### Study population and outcomes

We included patients above 18 years of age with a CRC diagnosis and date of surgery between May 1st 2001 and December 31st 2019 in both DCCG and DNPR and where the surgery date in DCCG is matched by the date in DNPR. The outcome of interest was all-cause mortality within 30 and 90 days and 1 year after CRC surgery.

### Statistical analyses

Each data source was transformed into the Observational Medicine Outcomes Network Common Data Model (OMOP-CDM) v5.3 and subsequently merged [25]. The OMOP-CDM structure enables the use of open-source analysis tools provided by the Observational Health Data Science and Informatics community (OHDSI) [26].

The initial index date was set to the date of surgery, and times-at-risk were set to 30 days, 90 days, and 1 year in order to assess different parts of the patient trajectory with varying impact of the surgical treatment on the individual survival points covering both immediate and more prolonged impact of surgery.

Data was divided into a training set consisting of a random 75% of patients for model development and 25% of patients were used for internal validation. Threefold cross-validation was used for hyperparameter optimization. When considering the possibility for missing data, OMOP-CDM requires sex and age, which is present for all records. However, for all other fields, there may be missing data. In DCCG, some covariates are mutually exclusive and thus can be separated from missing values such as deficient mismatch repair (dMMR) and proficient mismatch repair (pMMR). In the remaining data sources, however, distinguishing between no record or missing record is not possible due to the nature of data capture from EHR and longitudinal data collection, for instance if there was no record of a specific diagnosis, it was not present in the specific patient. Categorical variables such as procedures and diagnoses were stored by one-hot encoding, causing both negative values and missing data for categorical variables to be interpreted as zero by the model. Missing continuous values were interpreted as NA and as such the record of a missing continuous variable would not be taken into consideration [25, 27, 28]. Apart from age, continuous variables were almost exclusively biochemical results, especially from blood samples.

We trained a Least Absolute Shrinkage and Selection Operator (LASSO) logistic regression model for 30-day, 90-day, and 1-year postoperative mortality [29–33]. The LASSO logistic regression generally performs well with rare outcomes or sparse data. The advantage of LASSO logistic regression is that the model is provided with all covariates above a certain amount, which we predefined as 0.1% of records, from the source data and automatically selects covariates, which are associated with the outcome and thus shrinks tens of thousands of covariates to only a fraction of relevant covariates. Performance of models was assessed using area under the receiver operating characteristic curve (AUROC) and area under precision-recall curve (AUPRC) for assessment of discrimination including calculating the ratio between incidence and AUPRC, and calibration intercept, calibration slope, and calibration-in-the-large for assessment of calibration. Visual representation of receiver operating characteristic, precision-recall curves, and calibration plots are reported and assessed visually [34].

In order to assess the performance of the model in different clinical scenarios related to acuteness and intent of surgery, we performed subgroup analysis by testing the model on subsets of patients, who underwent curative, palliative, emergency, and elective surgery, respectively. In the subgroup analyses, the model was trained on the full training set and subsequently validated on the different subsets of patients.

The tool used for covariate selection and model settings was ATLAS version 2.9.0. For model training, R v. 4.0.3 was used with the “PatientLevelPrediction” package v. 4.3.7. Reporting of outcomes of this study adheres to the Transparent Reporting of a multivariable prediction model for Individual Prognosis Or Diagnosis (TRIPOD) guidelines [35].

## Results

### Participants

**Fig. 1:**
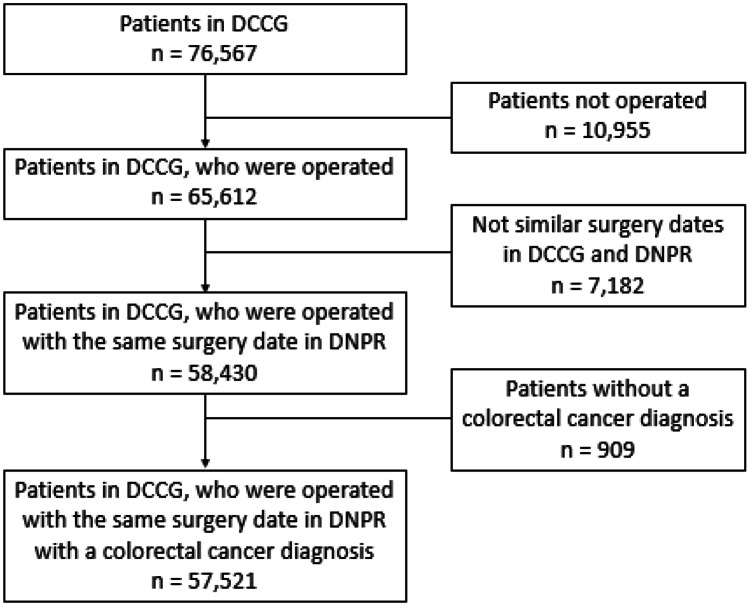
Flowchart of patient inclusion. DCCG Danish Colorectal Cancer Group database, DNPR Danish National Patient Register

**Fig. 2:**
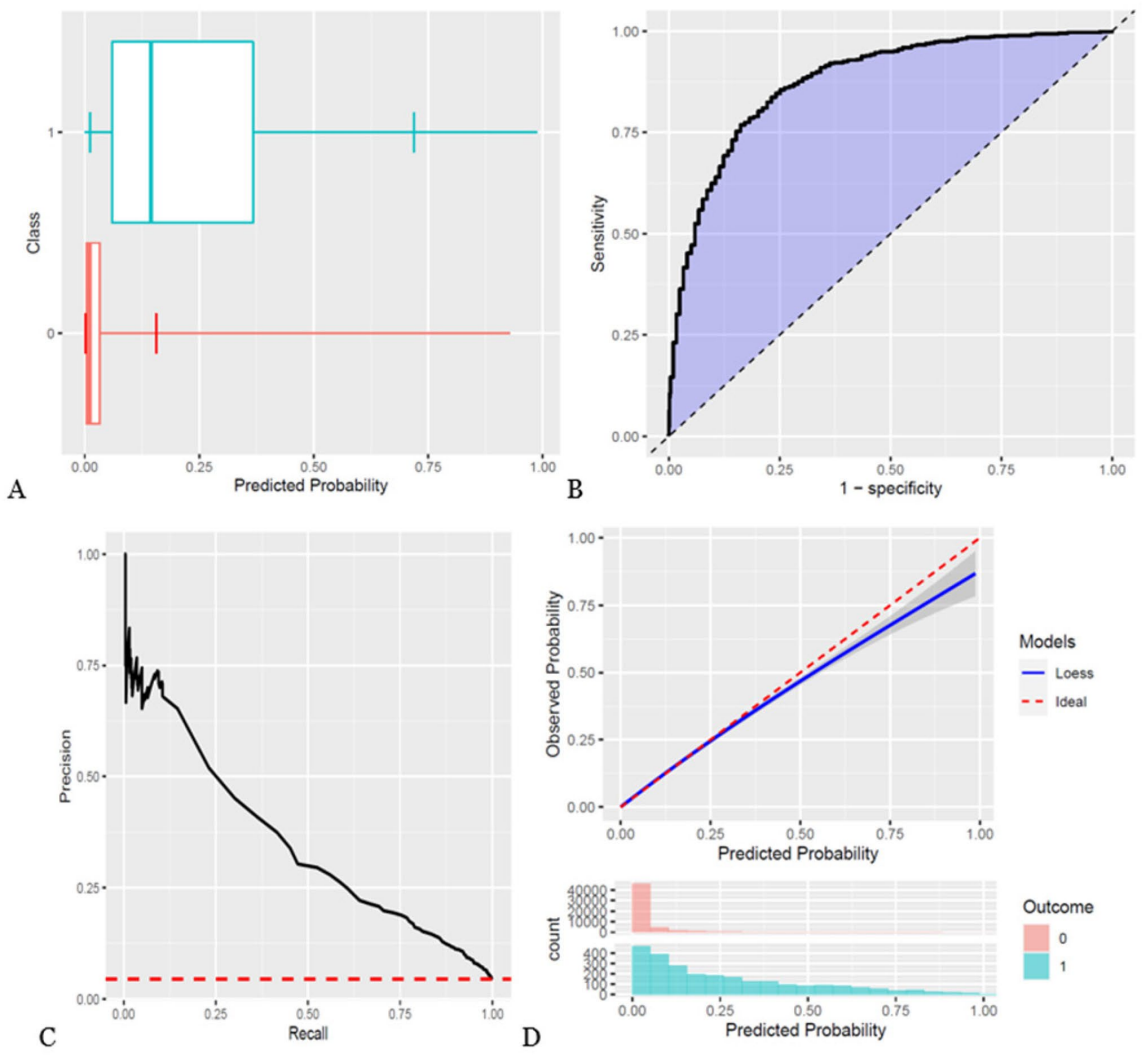
Study outcomes for 30-day mortality. **A** Boxplot of predicted risk for patients, who died (blue) and patients, who did not die (red). **B** Receiver operation characteristic (ROC) curve for 30-day mortality. **C** Precision-recall curve for 30-day mortality. **D** Calibration plot for 30-day mortality. Blue color is cases (mortality within the time at risk); red color is patients, who did not die within the time at risk

**Fig. 3:**
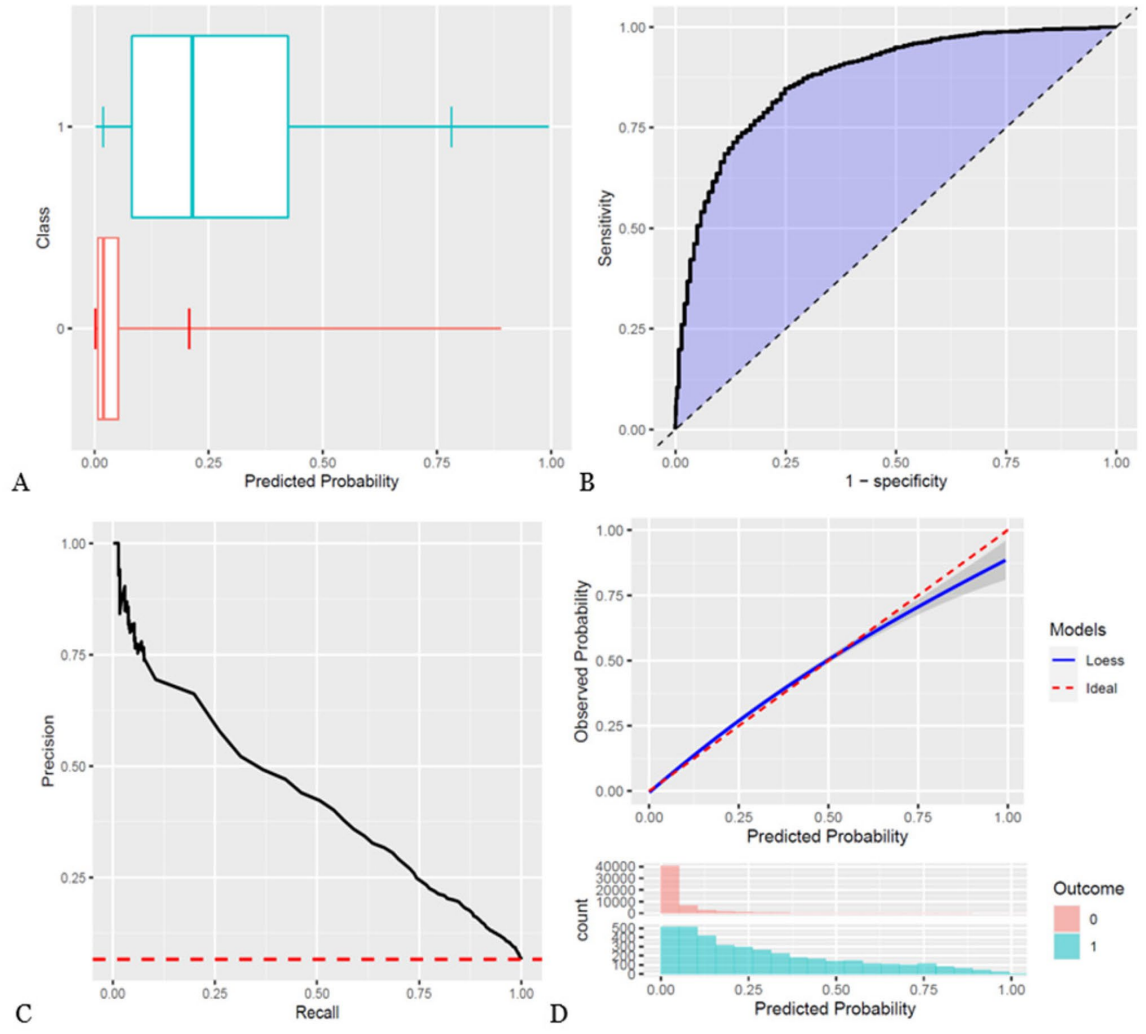
Study outcomes for 90-day mortality. **A** Boxplot of predicted risk for patients, who died (blue) and patients, who did not die (red). **B** Receiver operation characteristic (ROC) curve for 90-day mortality. **C** Precision-recall curve for 90-day mortality. **D** Calibration plot for 90-day mortality. Blue color is cases (mortality within the time at risk); red color is patients, who did not die within the time at risk

**Fig. 4:**
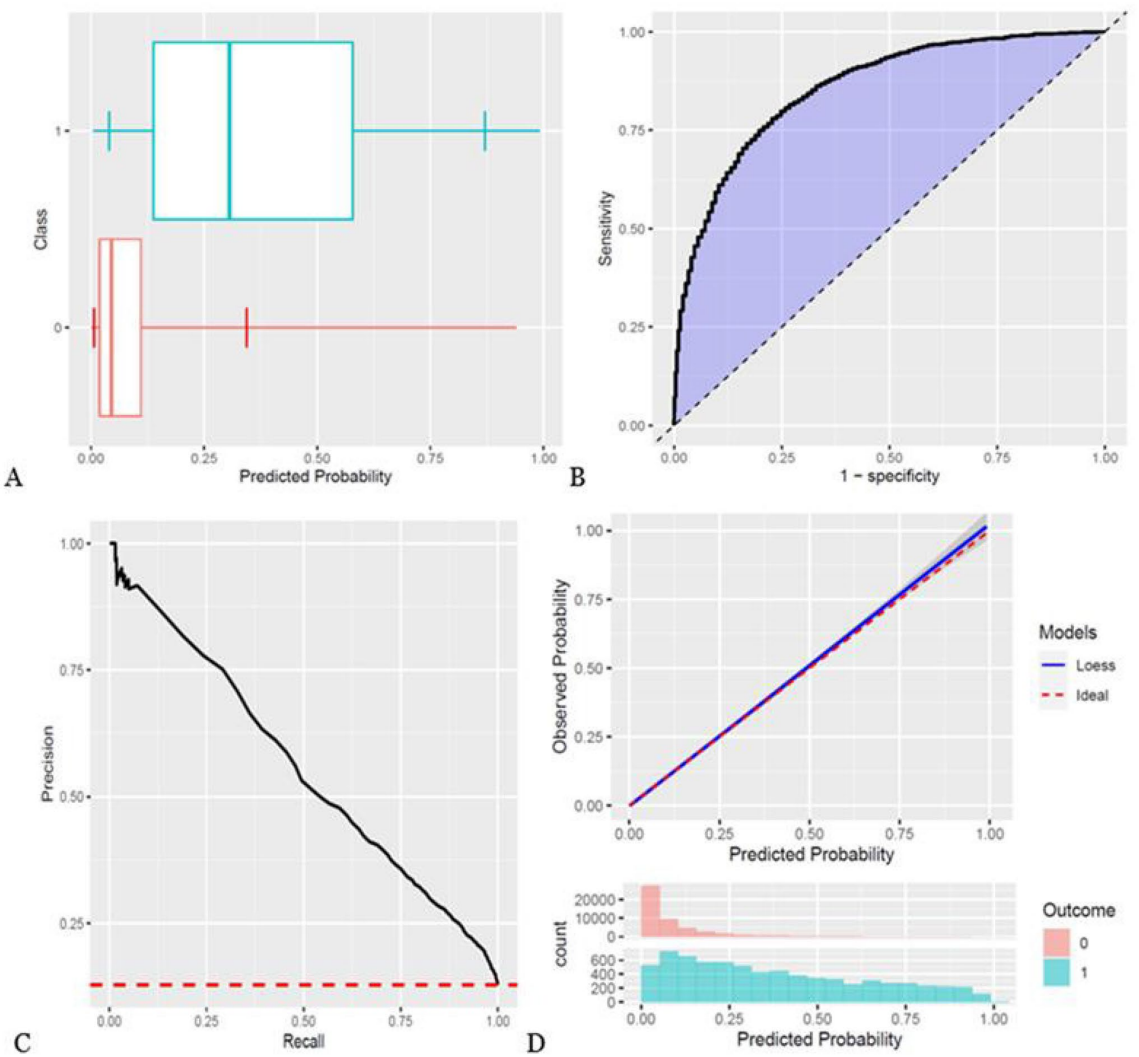
Study outcomes for 1-year mortality. **A** Boxplot of predicted risk for patients, who died (blue) and patients, who did not die (red). **B** Receiver operation characteristic (ROC) curve for 1-year mortality. **C** Precision-recall curve for 1-year mortality. **D** Calibration plot for 1-year mortality. Blue color is cases (mortality within the time at risk); red color is patients, who did not die within the time at risk

A total of 65,612 patients underwent surgery for CRC from 2001 to 2019 in DCCG. Of these patients, 57,521 patients were included in the study cohort (Fig. 1). This cohort consisted of 48.9% female patients, with a median age of 72 years. The remaining patient characteristics are found in Table 1 and boxplots showing the predicted risk in the mortality and no mortality groups are found in Figs. 2A, 3A, and 4A. Incidences of mortality were 4.48%, 6.64%, and 12.8% for 30-day, 90-day, and 1-year mortality, respectively.

**Table 1:** Demographic and clinical parameters for patients included in the cohort

**Characteristic**	**Count (*n*** **= 57,521)**	**%**
**Number of events**		
30-day mortality incidence	2579	4.48
90-day mortality incidence	3824	6.65
1-year mortality incidence	7383	12.8
**Basic information**		
Male	30,668	53.3
Female	26,890	46.7
Age (median, IQR)	Median 71	IQR* 64–75
**Lifestyle and comorbidity**		
ASA* score		
1	12,682	22.0
2	30,267	52.6
≥3	13,018	22.6
Missing	1591	2.8
WHO* performance status		
0	13,545	23.5
1	4943	8.6
≥2	1972	3.4
Missing	37,098	64.5
**Charlson’s comorbidity index**		
0	36,749	63.8
1	9632	16.7
2	5743	10
≥3	5432	9.4
Missing	< 6	0
**Alcohol consumption per week**		
0 units	10,499	18.2
1–14 units	26,951	46.8
15–21 units	3528	6.1
≥22 units	3249	5.6
Missing	13,331	23.2
**Body mass index**		
≤18.5	1581	2.7
18.5–25	21,283	37.0
25–30	17,216	29.9
30–35	5733	10.0
≥35	1942	3.4
Missing	9803	17.0
**Smoking status**		
Smoker	9485	16.5
Previous smoker	19,367	33.6
Never smoked	17,531	30.5
Missing	11,175	19.4
**Cancer topography**		
Colon cancer	40,160	69.8
Rectum cancer	19,321	33.6
**Tumor specific details**		
T stage*		
T0	451	0.8
T1	9482	16.5
T2	10,256	17.8
T3	27,110	47.1
T4	8703	15.1
Missing	1556	2.7
N stage*		
N0	27,789	48.3
N1	12,514	21.7
N2	8700	15.1
N3	389	0.7
Missing	8166	14.2
M stage*		
M0	51,864	90.1
M1	8022	13.9
**Treatment details**		
Family history of malignant neoplasm of gastrointestinal tract	14,600	25.4
**Treatment intent**		
Curative	45,135	78.4
Palliative	2237	3.9
Compromised resection	406	0.7
Missing	9780	17.0

^*^IQR interquartile range, *ASA* American Society of Anesthesiology, *WHO* World Health Organization, *T stage* tumor size stage, *N stage* lymph node stage, *M stage* remote metastasis stage

### Model performance

The models contained 419, 561, and 581 covariates for 30-day, 90-day, and 1-year mortality, respectively. The models with all details can be viewed in supplementary Table 1.

The models showed discrimination of AUROC of 0.88, 0.878, and 0.861 (Table 2, Figs. 2B, 3B, and 4B) and AUPRC of 0.353, 0.431, and 0.559 for 30-day, 90-day, and 1-year mortality, respectively, where the incidence was 0.448, 0.665, and 0.128 (Table 2, Figs. 2C, 3C, and 4C). In terms of calibration, the models had calibration slopes of 0.96, 1.02, and 1.04, calibration intercepts of 0.02, 0.01, and 0.01, and calibration-in-the-large of 1.01, 0.99, and 0.99 for 30-day, 90-day, and 1-year mortality (Table 2, Figs. 2D, 3D, and 4D). Additionally, the visual assessment of performance in terms of the smooth calibration plots was excellent for 30-, 90-day, and 1-year mortality as can be viewed in Figs. 2D, 3D, and 4D.

**Table 2:** Cohort, calibration, and discrimination metrics for 30-, 90-, and 180-day post-operative mortality using LASSO logistic regression

	**Number of patients**	**Number of outcomes**	**Candidate covariates (included)**	**AUROC**	**AUPRC**	**Calibration in the large**	**Calibration slope**	**Calibration intercept**
**30-day mortality**	57,521	2579 (4.48%)	24,744 (419)	0.88	0.353	1.01	0.96	0.02
**90-day mortality**	57,521	3824 (6.65%)	24,744 (561)	0.878	0.431	0.99	1.02	0.01
**1-year mortality**	57,521	7383 (12.8%)	24,744 (581)	0.861	0.559	0.99	1.04	0.01

*AUROC* area under the receiver operating characteristic, *AUPRC* area under precision-recall curve

In subgroup analysis, the results from the general model including all patients operated for CRC were compared to smaller subgroups of patients undergoing elective surgery (*n* = 30,167), patients undergoing emergency surgery (*n* = 4279), patients undergoing palliative surgery (*n* = 1829), and patients undergoing curative surgery (*n* = 24,598). A comparison of all performance evaluation measures in each subgroup can be viewed in Table 3.

**Table 3:** Performance shown with discrimination and calibration evaluation methods in subgroup analysis

	**Cohort**	**Number of patients**	**Incidence of outcomes**	**AUROC**	**AUPRC (relative to incidence)**	**Calibration in the large**	**Calibration slope**	**Weak calibration intercept**	**Hosmer–Lemeshow intercept**
**30-day mortality**	**All surgeries**	57,521	4.48%	0.880	0.353 (7.8)	1.01	0.96	0.11	0.00
	**Elective**	51,695	3.1%	0.886	0.275 (8.9)	1.00	1.12	0.31	0.00
	**Emergency**	5691	17.1%	0.877	0.608 (3.6)	0.98	1.05	0.14	0.00
	**Curative**	45,135	3.0%	0.896	0.329 (11)	1.00	1.10	0.23	0.01
	**Palliative**	2251	15.0%	0.865	0.588 (3.9)	0.95	1.06	0.16	0.00
**90-day mortality**	**All surgeries**	57,521	6.65%	0.878	0.431 (6.4)	0.99	1.02	0.07	0.00
	**Elective**	51,695	4.7%	0.873	0.341 (7.1)	0.99	1.13	0.30	0.01
	**Emergency**	5691	23.6%	0.864	0.671 (2.8)	1.01	1.13	0.12	0.01
	**Curative**	45,135	4.5%	0.879	0.367 (8.3)	1.01	1.11	0.24	0.00
	**Palliative**	2251	25.1%	0.829	0.652 (2.6)	1.03	0.99	0.05	0.01
**1-year mortality**	**All surgeries**	57,521	12.9%	0.861	0.559 (4.3)	0.99	1.04	0.06	0.00
	**Elective**	51,695	10.1%	0.854	0.469 (4.6)	1.00	1.10	0.16	0.01
	**Emergency**	5691	37.8%	0.852	0.781 (2.1)	1.00	1.13	0.05	0.02
	**Curative**	45,135	9.0%	0.850	0.429 (4.8)	0.99	1.07	0.11	0.00
	**Palliative**	2251	46.6%	0.812	0.788 (1.7)	0.97	1.03	0.08	0.01

*AUROC* area under the receiver operating characteristic, *AUPRC* area under precision-recall curve

## Discussion

In summary, we used observational health data from national registers to train models by a machine learning algorithm, which identify the most important predictors for 30-day, 90-day, and 1-year mortality following colorectal cancer surgery. The model showed overall good performance, especially for 1-year mortality (Fig. 4). In the subgroup analysis comparing the general population with subgroups of patients undergoing curative, palliative, emergency, or elective surgery, we consistently found good performance comparable to the larger study population in each subgroup, showing that the model can be used in multiple circumstances.

The aim of the study was to create a model to binary classify mortality during different time windows based on a high granular national dataset. The high number of candidate features of the model versus the relative few patients can cause a regression model to have a high variability if using a least squares fitting procedure; however, statistical learning methods within ML can often decrease the overall error of the predictions, by reducing the variability at the cost of a negligible bias increase [36]. A common concern when creating such prediction models is a “black box” effect, where the relationship between the input parameters and the output becomes opaque, which might cause clinicians to trust and act on the output [37]. The study uses the LASSO logistic regression as a statistical learning method, which imposes a restriction on the coefficient estimates, which is otherwise identical to ordinary least squares. This method keeps the intrinsic interpretability of regression-based models and performs variable selection, resulting in sparse models, which further aids interpretability [36, 38]. While the model itself is interpretable and does not contain any higher order polynomials or interaction terms, the number of covariates included in all models, in clinical implementation might benefit for solutions designed to communicate the relationship between input and output of the model [38].

Other risk assessment tools currently exist, but are not routinely used in the clinical setting, and tend to perform less impressively on internal validation sets [39–41]. Additionally, most prediction studies tend to mainly report AUROC, but not other performance metrics, especially calibration metrics [42]. In this study, we report a range of graphical and numerical discrimination and calibration measures with full transparency, which also lives up to the reporting guidelines in the TRIPOD statement [35].

Using prediction models for clinical risk assessment is known to have implementation challenges, partly because of suboptimal introduction to the clinicians, which in some cases have been seen to cause lack of trust in the prediction [43, 44]. When interpretability comes into question, it is, however, important to note that increasing explainability of the model may reduce the performance and clinical applicability [45]. The data-driven covariate inclusion means that some included covariates may seem clinically unrelated to the outcome. The more traditional approach is the pre-selection of variables based upon clinical knowledge and existing known causal relationships. By doing a pre-selection, the potential search space for the model is vastly reduced and removes the potential for finding new associations that can be predictive of the outcome. As such this method, while providing models with a high explainability, may sacrifice the potential for better performance. This study does not report positive-predictive value, negative-predictive value, sensitivity, and specificity, since they require a threshold for high risk. However, establishment of a high risk is greatly dependent on the patient phenotype and having high or low risk as result of a prediction rather than a number, may lead to the model being used as a deciding factor, and not clinical decision support.

One of the main strengths of this study is the use of high-quality observational health databases with national coverage, high rate of completeness [19–21], high granularity in data, and a combination of differently focused data sources. This yields many patients in the cohort, which provides more data for model training but also reduces selection bias from variation in local practices. The merging of different health databases with a different focus, cancer-specific trajectory data, admission-specific data, prescription medicine data, or laboratory results data, also provides the most comprehensive data coverage related to each patient, which is necessary in order to create full patient phenotypes. An additional strength is the testing of the model in a subgroup analysis of different surgical settings, acute, elective, palliative, and curative, where the model showed equal performance in all subgroups. This additional internal validation step supports that the model provides reliable risk predictions for patients in multiple different settings.

Denmark is known to have observational health databases with high coverage, but other countries such as the Netherlands (for instance Netherlands Cancer Registry provided by Netherlands comprehensive cancer organization [46]) and Norway (for instance Cancer Registry of Norway [47]) show similar datasets and coverage to the Danish databases. This means that models likely can be used in different countries with similar datasets. Additionally, the models are trained to use the provided covariates, meaning that models can be used on incomplete datasets.

The study evaluates the performance of prediction models developed using data harmonized to the OMOP-CDM based on several national Danish registers. The use of open-source tools and the overall model development allows other data holders to convert their data to the OMOP format and train their own prediction model. The covariates with the largest positive and negative covariates are presented in the supplementary.

The data-driven approach meant that all available covariates could potentially be included in the model development. The LASSO logistic regression selects covariates for the model based on their impact on the model and excludes covariates with no effect on the prediction. The use of this data-driven covariate selection often leads to many included covariates, which means that these models utilized 419, 561, and 581 one-hot encoded covariates for prediction, which is infeasible for clinical implementation. A step towards making the number of variables smaller is by grouping them into categories or phenotypes, which will make the model more clinically manageable, a so-called parsimonious model [48, 49]. This would however potentially decrease the performance of the model or may introduce bias, when covariates are selectively grouped. The prospect of parsimonious models is interesting for the next steps of clinical implementation but will require further research into how the number of covariates can be shrunk with minimal model impact. In addition, external validation with other colorectal cancer research groups with an OMOP-CDM would be an important future step to ensure that models are clinically applicable internationally.

The limitations of this study included the fact that external validation was not yet possible and that the models have many included covariates, where further research is required.

However, the strengths of the study was also considerable the two largest being the use of national health databases with good coverage rather than single or few centers and the very large sample size of over 50,000 patients. Additional strengths worth mentioning are the use of only preoperative covariates, internal validation where the model is tested on a separate group of records not used for training the model, and with reporting of multiple measurements of performance including calibration and discrimination, which is rarely provided in similar detail.

## Conclusion

We found that the combination of multiple nationwide databases for patients with CRC allowed for development of high performing prediction models for mortality with great calibration and discrimination. By only including preoperative covariates, the models are usable in the treatment planning phase and may assist clinicians with optimizing an individualized approach to colorectal cancer treatment.

## Supplementary Information

Below is the link to the electronic supplementary material.

Supplementary file1 (DOCX 31 KB)

## Data Availability

Data consisted of the Danish Colorectal Cancer Group database (DCCG), which was provided by the Regional Clinical Quality Program (RKKP), and the Danish National Patient Register (DNPR), Register of Laboratory Results for Research (RLRR), and the National Prescription Registry (NPR) provided by the Danish Health Data Authorities. Accessibility to data is dependent on approval from these regulatory bodies and appropriate applications.
