# Supplementary material for: Prediction of 30-day, 90-day, and 1-year mortality after colorectal cancer surgery using a data-driven approach
Source: Int J Colorectal Dis. 2024 Feb 29;39(1):31. doi: 10.1007/s00384-024-04607-w (PMC10904562; doi:10.1007/s00384-024-04607-w)
Supplement: Supplementary file 1 — Supplementary file1 (DOCX 31 KB) [file 384_2024_4607_MOESM1_ESM.docx]

# Supplementary material: Model design

## Target cohort definition

**Cohort entry event**

Patients having a curatively intended procedure defined under “List of curatively intended surgeries” in Danish Colorectal Cancer Group database (DCCG) and 0 days before and 0 days after that procedure date in Danish National Patient Register (DNPR)

OR

Patients having a palliatively inteded intended procedure defined under “List of palliatively intended surgeries” in Danish Colorectal Cancer Group database (DCCG) and 0 days before and 0 days after that procedure date in Danish National Patient Register (DNPR)

**Additional inclusion criteria**

Registration of a colorectal cancer diagnosis maximum 180 days before date of surgery and maximum 60 days after surgery (Concept ID: ‘Primary Malignant Neoplasm of Rectum’, 74582, AND ‘Primary Malignant Neoplasm of Colon’, 197500)

## Outcome cohort definition

## Model settings

## Time at risk

30 days from date of surgery

90 days from date of surgery

365 days from date of surgery

*Patients*

## Concept lists

**List of curatively intended procedures**

| Concept name | Additional information | Concept ID |
| --- | --- | --- |
| Abdominoperineal resection | Include all descendants | 4144721 |
| Anterior resection of rectum with colostomy | Include all descendants | 4233085 |
| EMR (endoscopic mucosal resection) of gastrointestinal tract | Include all descendants | 36712676 |
| Excision of colon | Include all descendants | 4079713 |
| Excision of rectal tumor, transanal approach; including muscularis propria (ie, full thickness) | Include all descendants | 40756915 |
| Extended right hemicolectomy | Include all descendants | 4017464 |
| Ileocolic resection | Include all descendants | 4078310 |
| Left colectomy | Include all descendants | 4219780 |
| Resection of rectum | Include all descendants | 4226983 |
| Right colectomy | Include all descendants | 4133412 |
| Sigmoid colectomy | Include all descendants | 4225427 |
| Sigmoid colectomy and colostomy | Include all descendants | 4146634 |
| Total abdominal colectomy with ileoproctostomy | Include all descendants | 4262950 |
| Total abdominal colectomy with proctectomy and ileostomy | Include all descendants | 4149741 |
| Total colectomy and ileostomy | Include all descendants | 4145225 |
| Transverse colectomy | Include all descendants | 4097958 |

**List of palliatively intended procedures**

| Concept name | Additional information | Concept ID |
| --- | --- | --- |
| Exploratory diagnostic procedures | Include all descendants | 4012522 |
| Endoscopic insertion of permanent colonic stent | Include all descendants | 4190934 |
| Colostomy | Include all descendants | 4162987 |

**Concepts to exclude**

| Concept name | Additional information | Concept ID |
| --- | --- | --- |
| Acute pulmonary insufficiency following non-thoracic surgery |  | 4055650 |
| Adjuvant therapy |  | 35803584 |
| Alcohol consumption per week | Replaced by custom concept | 2147483463 |
| American Society of Anesthesiologists physical status classification | Replaced by custom concept | 4159411 |
| Anastomosis of intestine |  | 4128868 |
| Anastomosis orientation: Other |  | 2147483485 |
| Anastomosis technique – Handsewn |  | 2147483484 |
| Anastomosis technique – intraluminal stapler |  | 2147483483 |
| Anastomosis technique - other |  | 2147483482 |
| Anastomotic leak, anastomosis kept |  | 2147483139 |
| Anastomotic leak, anastomosis removed |  | 2147483138 |
| Antibiotic prophylaxis |  | 4226249 |
| Assistant surgeon |  | 42739595 |
| Assistant surgeon: Certified colorectal surgeon |  | 2147483478 |
| Assistant surgeon: No assisting surgeon |  | 2147483477 |
| Assistant surgeon: Not a physician |  | 2147483476 |
| Assistant surgeon: Resident surgeon |  | 2147483474 |
| Assistant surgeon: Surgeon/surgical gastroenterologist |  | 2147483475 |
| Bladder excision |  | 4029571 |
| Bleeding |  | 437312 |
| Blood loss [volume] measured |  | 21493943 |
| Body height |  | 3036277 |
| Body weight |  | 3025315 |
| BRAF gene mutation |  | 35976956 |
| Cadaver donor |  | 40268267 |
| Cadaver donor |  | 4162719 |
| Capecitabine |  | 1337620 |
| Cardiac arrest with successful resuscitation |  | 4120088 |
| Central ligation of ileocolic artery |  | 2147483446 |
| Central ligation of middle colic artery |  | 2147483444 |
| Charlson Comorbidity Index | Replaced by custom concept | 42538860 |
| Cholecystectomy |  | 4242997 |
| Clavien-Dindo complication scale | Replaced by custom concept | 37311607 |
| Combined chemotherapy and radiation therapy |  | 45766298 |
| Combined preoperative chemotherapy and radiation therapy | Replaced by custom concept | 4191719 |
| Complication associated with genitounrinary device |  | 43021248 |
| Complication of medical care |  | 440005 |
| Complication of procedure |  | 442019 |
| Conducted additional excision |  | 2147483232 |
| Congestive heart failure as post-procedure complication of non-cardiac surgery |  | 762003 |
| Consequence of gastrointestinal anastomotic leak: anastomosis broken down |  | 2147483249 |
| Consequence of gastrointestinal anastomotic leak: anastomosis retained |  | 21474834248 |
| Contact person |  | 4323959 |
| Converted to multiport laparoscopy |  | 2147483462 |
| Creation of defunctioning ileostomy |  | 4297514 |
| Creation of defunctioning colostomy |  | 4142244 |
| Dead |  | 434489 |
| Death |  | 4306655 |
| Death of unknown case |  | 441413 |
| Deapest extent of tumor invasion in colorectal cancer specimen, resectate |  | 2147483280 |
| Dehiscence of anastomosis |  | 40490359 |
| Dehiscence of fascia |  | 40480451 |
| Dehiscence of internal surgical wound |  | 40482236 |
| Dehiscence of surgical wound |  | 136580 |
| Died in hospital |  | 4081765 |
| Distance from tumor to anal verge by rigid endoscopy | Replaced by custom concept | 2147483231 |
| Distance from tumor to anal verge on MRI | Replaced by custom concept | 2147483432 |
| Distance from tumor to closest margin |  | 3043442 |
| End-to-end anastomosis – action |  | 4117618 |
| End-to-side anastomosis – action |  | 4117961 |
| Excision of gastrocolic ligament |  | 4141457 |
| Excision of intra-abdominal mass |  | 4305331 |
| Excision of lesion of abdominal wall |  | 4253069 |
| Excision of lesion of peritoneum |  | 4195874 |
| Extracorporeal anastomosis |  | 2147483487 |
| Failed procedure using minimally invasive approach converted to open procedure |  | 46272989 |
| Finding of tumor invasion of peritoneal cavity |  | 2147483310 |
| Flourouracil |  | 955632 |
| Flush ligation of inferior mesenteric artery |  | 4216463 |
| Folinic acid-flourouracil-oxiliplatin regimen |  | 37208180 |
| Follow-up encounter |  | 4307024 |
| Follow-up visit |  | 4089050 |
| Gastrointestinal anastomotic leak |  | 4341249 |
| Gastrointestinal complication of procedure |  | 4178810 |
| Hemorrhage of rectum and anus |  | 197925 |
| Histopathology finding |  | 4095606 |
| Home |  | 4139502 |
| Hospital readmission |  | 4213258 |
| Imaging |  | 4180938 |
| Indication for procedure |  | 4197035 |
| Injury during surgery, location not specified |  | 2147483456 |
| Injury of bladder during surgery |  | 37117795 |
| Injury of gallbladder during surgery |  | 37395689 |
| Injury of spleen during surgery |  | 46272915 |
| Injury of colon during surgery |  | 2147483454 |
| Injury of duodenum during surgery |  | 2147483455 |
| Injury of liver during surgery |  | 37017028 |
| Injury of pancreas during surgery |  | 2147483453 |
| Injury of sacral vein during surgery |  | 2147483452 |
| Injury of small intestine during surgery |  | 2147483451 |
| Injury of ureter during surgery |  | 2147483450 |
| Injury of urethra during surgery |  | 2147483449 |
| Injury of vagina during surgery |  | 2147483448 |
| Injury of viscus during surgery |  | 4309340 |
| Intactness of mesorectal specimen |  | 4256886 |
| Intestinal obstruction |  | 193518 |
| Intestinal obstruction co-occurrent and due to decreased peristalsis |  | 46272242 |
| Intra-abdominal hemorrhage post-procedure |  | 4205675 |
| Intracorporeal anastomosis |  | 2147483486 |
| Intraoperative finding of liver metastasis |  | 2147483254 |
| Intraoperative finding of secondary malignant neoplasm of intra-abdominal organs |  | 2147483257 |
| Intraoperative finding of secondary malignant neoplasm, location not specified |  | 2147483256 |
| Intraoperative finding of secondary malignant neoplasm of peritoneum |  | 2147483255 |
| Intraoperative transfusion of blood product |  | 2147483261 |
| Intraoperative tumor fixation finding, tumor not removable |  | 2147483230 |
| Intraoperative tumor fixation finding, tumor removable |  | 2147483229 |
| KRAS gene mutations found [Identifier] in Colorectal cancer specimen by Molecular genetics method nominal |  | 36203353 |
| Leucovorin |  | 1388796 |
| Ligation of inferior mesenteric artery with preservation of ascending left colic artery |  | 4215699 |
| Ligation of left branch of middle colic artery |  | 2147483442 |
| Ligation of left colic artery |  | 2147483441 |
| Ligation of major artery of abdomen |  | 4201891 |
| Ligation of right branch of middle colic artery |  | 2147483443 |
| Ligation of sigmoid arteries |  | 2147483440 |
| Local recurrence of malignant tumor of colon |  | 4201482 |
| Loss of consciousness |  | 372448 |
| Low blood pressure |  | 317002 |
| Lymph nodes with metastases [#] in Cancer specimen |  | 37020577 |
| Lymphatic (small vessel) invasion by tumor present |  | 4217720 |
| Malignant neoplasm of colon and/or rectum |  | 36683531 |
| Mechanical complication of genitourinary device |  | 4006481 |
| Mechanical complication of genitourinary device, implant AND/OR graft |  | 442012 |
| Medical examination for suspected condition |  | 4064522 |
| Mesorectal specimen completely intact |  | 4248805 |
| Multidisciplinary care conference |  | 4296791 |
| Neoadjuvant therapy | Replaced by custom concept | 35804141 |
| No evidence of |  | 4211787 |
| No tumor present in specimen |  | 4287029 |
| NRAS gene mutation |  | 2147483066 |
| NRAS gene mutations found [Identifier] in Colorectal cancer specimen by Molecular genetics method nominal |  | 36203354 |
| Oral and rectal bowel preparation |  | 2147483241 |
| Oral bowel preparation |  | 2147483240 |
| Oxiliplatin |  | 1318011 |
| Pathologist report |  | 4023913 |
| Pathologist report, primary malignant tumor of cecum |  | 2147483279 |
| Pathologist report, primary malignant tumor of colon |  | 2147483278 |
| Pathologist report, primary malignant tumor of descending colon |  | 2147483277 |
| Pathologist report, primary malignant tumor of large intestine |  | 2147483276 |
| Pathologist report, primary malignant tumor of rectum |  | 2147483275 |
| Pathologist report, primary malignant tumor of sigmoid colon |  | 2147483274 |
| Pathologist report, primary malignant tumor of the ascending colon |  | 2147483273 |
| Pathologist report, primary malignant tumor of the left colonic flexure |  | 2147483272 |
| Pathologist report, primary malignant tumor of the right colonic flexure |  | 2147483271 |
| Pathologist report, primary malignant tumor of transverse colon |  | 2147483270 |
| Pathologist report: Cancer type, primary malignant tumor of colon |  | 2147483375 |
| Pathologist report: Cancer type, primary malignant tumor of colon or rectum |  | 2147483374 |
| Pathologist report: Cancer type, primary malignant tumor of rectum |  | 2147483373 |
| Patient fulfills criteria for readmission eligibility |  | 2147483502 |
| Patient fulfills criteria for recurrence eligibility |  | 2147483503 |
| Perforation |  | 4263947 |
| Perforation of large intestine |  | 4263947 |
| Perforation of large intestine , intraoperatively |  | 2147483264 |
| Perforation of large intestine during stenting < 36 hour prior |  | 2147483263 |
| Perforation of large intestine in non-tumor bearing bowel segment |  | 2147483269 |
| Perforation of large intestine in tumor bearing bowel segment |  | 2147483268 |
| Perforation of large intestine in unknown bowel segment |  | 2147483267 |
| Perineural invasion by tumor present |  | 4183085 |
| Peripheral ligation of ileocolic artery |  | 2147483445 |
| Peripheral ligation of superior rectal artery |  | 2147483439 |
| Permanent colostomy | Replaced by custom concept | 4224467 |
| Permanent ileostomy | Replaced by custom concept | 4279534 |
| pM1 category |  | 4033703 |
| pN0 category |  | 4071446 |
| pN1 category |  | 4163665 |
| pN1 category, however several categories designated |  | 2147483400 |
| pN1 category, however several stages designated |  | 2147483304 |
| pN1a category |  | 40482826 |
| pN1b category |  | 40482360 |
| pN1c category |  | 40483202 |
| pN2 category |  | 4035791 |
| pN2 category, however several categories designated |  | 2147483399 |
| pN2 category, however several stages designated |  | 2147483303 |
| pN2a category |  | 40483686 |
| pN2b category |  | 40481863 |
| pNX category |  | 4182531 |
| Positron emission tomography |  | 4305790 |
| Postoperative anastomotic leak grade |  | 2147483250 |
| Postoperative complication |  | 4300243 |
| Postoperative complication of anastomosis or stoma of gastrointestinal tract |  | 2147483136 |
| Postoperative intestinal obstruction |  | 4340367 |
| Postoperative medical complication |  | 2147483246 |
| Postoperative medical complication: Other |  | 2147483247 |
| Postoperative state |  | 438485 |
| Postoperative surgical complication |  | 2147483252 |
| Postoperative surgical complication: Other |  | 2147483253 |
| Postprocedural abscess |  | 36712819 |
| Postprocedural infection |  | 36712821 |
| Postprocedural intraabdominal abscess |  | 36712818 |
| Postprocedural respiratory disorders |  | 4024127 |
| Postprocedural state finding |  | 444239 |
| Preparation of bowel for procedure |  | 4074335 |
| Primary surgeon: Certified colorectal surgeon |  | 2147483473 |
| Primary surgeon: Nurse |  | 2147483472 |
| Primary surgeon: Other |  | 2147483471 |
| Primary surgeon: Resident surgeon |  | 2147483468 |
| Primary surgeon: Surgeon / Surgical gastroenterologist |  | 2147483470 |
| Primary surgeon: Unknown, procedure performed at other department |  | 2147483469 |
| pT0 category |  | 4218531 |
| pT1 category |  | 4207195 |
| pT2 category |  | 4217143 |
| pT2 category, however several categories designated |  | 2147483297 |
| pT3 category |  | 4234703 |
| pT3 category, however several categories designated |  | 2147483296 |
| pT4 category |  | 4266213 |
| pT4 category, however several categories designated |  | 2147483295 |
| pT4a category |  | 40482367 |
| pT4b category |  | 40483194 |
| pTX category |  | 4182266 |
| Pulmonary insufficiency following surgery |  | 4024117 |
| Radiation oncology AND/OR radiotherapy | Replaced by custom concept | 4029715 |
| Radical excision |  | 4267296 |
| Radiofrequency ablation of lesion of liver |  | 40491520 |
| Re-operation: Exploratory laparotomy due to suspicion of complication, but no complication found |  | 2147483251 |
| Rectal bowel preparation |  | 2147483239 |
| Rectal irrigation |  | 4102150 |
| Rectal tumor distance from anal verge by MRI | Replaced by custom concept | 2147483223 |
| Rectal tumor distance from anal verge by stiff rectoscope |  | 2147483224 |
| Recurrent tumor |  | 4097297 |
| Referral to oncologist |  | 4084352 |
| Regional lymph nodes examined [#] Specimen |  | 3013500 |
| Resection plane of colon, intramesocolic |  | 21474833309 |
| Resection plane of colon, mesocolic |  | 21474833308 |
| Resection plane of colon, muscularis propria |  | 21474833307 |
| Resection plane of colon, extra-levator plane |  | 21474833305 |
| Resection plane of colon, Sphincteric plane |  | 21474833306 |
| Residual tumor stage finding |  | 4300139 |
| Residual tumor stage R0 |  | 4121182 |
| Residual tumor stage R1 |  | 4175704 |
| Residual tumor stage R2 |  | 4081832 |
| Satellite nodules [#] in Colorectal cancer specimen by Light microscopy |  | 36203352 |
| Satellite nodules [Presence] in Colorectal cancer specimen |  | 42527709 |
| Side-to-end anastomosis - action |  | 4136008 |
| Side-to-side anastomosis |  | 4117964 |
| Size Tumor |  | 3018102 |
| Small intestine excision |  | 4030148 |
| Support |  | 4055254 |
| Surgeon primary [Identifier] |  | 3006395 |
| Surgery |  | 4121697 |
| Surgery performed by specialist colorectal surgeon |  | 2147483233 |
| Surgical follow-up |  | 4079646 |
| Temporary colostomy | Replaced by custom concept | 4297515 |
| Temporary ileostomy | Replaced by custom concept | 4017329 |
| Transfusion of blood product |  | 4024656 |
| TRUS T category cannot be assessed |  | 2147483220 |
| Tumor budding |  | 3195917 |
| Tumor Deposits |  | 35918596 |
| Tumor invades abdominal cavity structure |  | 4233769 |
| Tumor invasion by direct extension from organ of origin to adjacent organ |  | 4257540 |
| Tumor invasion finding |  | 4159956 |
| Tumor penetrated serosa, perforation present |  | 4161166 |
| Tumor regression grade a.m. Mandard |  | 2147483329 |
| Under care of colorectal surgeon |  | 4124095 |
| Under care of oncologist |  | 44813828 |
| Venous (large vessel) extramural invasion by tumor present |  | 4241061 |
| Venous (large vessel) extramural invasion by tumor present, both macroscopically and microscopically |  | 2147483323 |
| Venous (large vessel) extramural invasion by tumor present, microscopically |  | 2147483322 |
| Venous (large vessel) intramural invasion by tumor present |  | 4241188 |
| Venous (large vessel) invasion by tumor present |  | 4184072 |
| Ward |  | 4023217 |
| WHO performance status scale | Replaced by custom concept | 4162588 |
| ypN0 category |  | 2147483398 |
| ypN1 category |  | 2147483397 |
| ypN1b category |  | 2147483300 |
| ypN1c category |  | 2147483396 |
| ypN2 category |  | 2147483395 |
| ypNx category |  | 2147483394 |
| ypT0 category |  | 2147483393 |
| ypT1 category |  | 2147483392 |
| ypT2 category |  | 2147483391 |
| ypT2 category, however several categories designated |  | 2147483390 |
| ypT3 category |  | 2147483389 |
| ypT3 category, however several categories designated |  | 218647483388 |
| ypT4 category |  | 2147483387 |
| ypT4 category, however several categories designated |  | 21474833 |
| ypTx category |  | 2147483385 |

## High covariate value variables (20 out of 245 positive covariate values)

| Covariate name | Covariate value | Covariate count | Covariate mean | Covariate standard deviation |
| --- | --- | --- | --- | --- |
| Pulmonary edema | 1.76007 | 45 | 0.012837 | 0.107935 |
| Sepsis | 0.925131 | 406 | 0.066752 | 0.222976 |
| Age group: 90-94 | 0.912581 | 728 | 0.069748 | 0.182201 |
| Pneumonitis due to inhaled substance | 0.765249 | 100 | 0.021823 | 0.134261 |
| Age group: 85-89 | 0.731065 | 2696 | 0.18742 | 0.251606 |
| Dobutamine | 0.682698 | 98 | 0.023534 | 0.142372 |
| Age group: 95-99 | 0.639054 | 88 | 0.013265 | 0.094318 |
| ASA Score 4 | 0.624146 | 805 | 0.109542 | 0.273614 |
| Cerebrovascular accident | 0.577825 | 280 | 0.027813 | 0.11521 |
| Age group: 80-84 | 0.484208 | 4755 | 0.24647 | 0.213661 |
| Renal failure syndrome | 0.434572 | 110 | 0.01626 | 0.103889 |
| Bacterial pneumonia | 0.434533 | 215 | 0.020967 | 0.098738 |
| Emergency operation | 0.3127 | 4279 | 0.3757 | 0.47097 |
| MX category | 0.29015 | 10386 | 0.28199 | -0.031 |
| ASA Score 3 | 0.23757 | 8208 | 0.47796 | 0.39734 |
| Transverse colostomy | 0.28601 | 530 | 0.05648 | 0.17243 |
| Atrial arrhythmia | 0.23579 | 3837 | 0.22465 | 0.23606 |
| Chronic obstructive pulmonary disease | 0.21685 | 1545 | 0.09243 | 0.14566 |
| Laparotomy | 0.1827 | 19353 | 0.82499 | 0.45339 |

## Low covariate value variables (20 out of 231 negative covariate values)

| Covariate name | Covariate value | Covariate count | Covariate mean | Covariate standard deviation |
| --- | --- | --- | --- | --- |
| Endoscopic procedure | -0.89063 | 1275 | 0.007702 | -0.14739 |
| ASA Score 1 | -0.78645 | 7148 | 0.042362 | -0.38365 |
| Age group: 50-54 | -0.57379 | 1542 | 0.007702 | -0.17249 |
| Never smoked tobacco | -0.44142 | 9550 | 0.140779 | -0.25501 |
| Varicose veins of lower extremity | -0.4211 | 2360 | 0.041934 | -0.08708 |
| Age group: 55-59 | -0.38343 | 2591 | 0.020967 | -0.18977 |
| Local macroradical excision of colorectal tumor | -0.38028 | 9657 | 0.103979 | -0.34366 |
| Colostomy present | -0.34238 | 914 | 0.007702 | -0.10798 |
| ASA Score 2 | -0.33984 | 17245 | 0.306376 | -0.30396 |
| Endoscopic Mucosa Resection | -0.318 | 1275 | 0.007702 | -0.14739 |
| Age group: 60-64 | -0.30988 | 3778 | 0.041078 | -0.19523 |
| Retention of urine | -0.29488 | 361 | 0.005563 | -0.04114 |
| Curative procedure intent | -0.28375 | 24598 | 0.50107 | -0.33764 |
| Laparoscopic right hemicolectomy | -0.28265 | 3130 | 0.033376 | -0.1786 |
| Elective | -0.27435 | 32787 | 0.864784 | -0.22548 |
| Ex-cigarette smoker | -0.26203 | 11066 | 0.207531 | -0.19484 |
| Admission to department | -0.25767 | 1988 | 0.068464 | 0.034231 |
| Female | -0.2572 | 16890 | 0.463415 | -0.03856 |
| Alcohol consumption per week, 1-14 units per week | -0.25082 | 15077 | 0.216517 | -0.36493 |
| Budesonide 0.2 MG/ACTUAT Inhalation Powder | -0.24331 | 1969 | 0.052204 | -0.01593 |
